# Exploratory study of handwriting disorders in school-aged children for a better nosography

**DOI:** 10.1192/j.eurpsy.2023.364

**Published:** 2023-07-19

**Authors:** C. Lopez, L. Vaivre-Douret

**Affiliations:** ^1^INSERM Unit 1018-CESP, Faculty of Medicine, University of Paris-Saclay, UVSQ, Villejuif; ^2^Faculty of Health, Department of Medicine Paris Descartes, University of Paris Cité; ^3^ Chair in Clinical Neurodeveloppmental Phenotyping, Institut Universitaire de France (IUF); ^4^Department of Endocrinology, IMAGINE Institute of Necker-Enfants Malades hospital; ^5^Department of Child Psychiatry, Necker-Enfants Malades Hospital, AP-HP.Centre, Paris, France

## Abstract

**Introduction:**

Handwriting disorders (HDs) are prevalent in elementary school children, but their nature is poorly understood. Moreover, the diagnosis of dysgraphia is often too quickly concluded on a single assessment.

**Objectives:**

In the present study, we aimed to use a transdisciplinary assessment approach. We looked for to provide objective data to better understand the nature and aetiology of HDs.

**Methods:**

27 school aged children with HDs aged 6-11 years were included in the study and were compared to typically developing children. They performed a normed prescriptural task of copying cycloid loops. Postural and gestural inter-segmental coordination of arm movements were recorded with two video cameras allowing 2D reconstruction of the gesture. Spatial/temporal kinetic and kinematic measures were recorded with a digital pen. All children underwent normed and standardized clinical assessments of neuropsychomotor, neuropsychological and oculomotor functions. The handwriting test (BKK) were used.

**Results:**

Handwriting disorders seem very heterogeneous. However, there is a significantly poorer gestural of inter-segmental coordination and of kinetic/kinematic performances of the tracings in HDs. Furthermore, it was possible to highlight three levels of HDs: mild HD not detected by the BHK test (26% of children), moderate HD with the BHK (33%), dysgraphia identified by the BHK (41% of children). The mild nature of HDs not dectected by the BHK seems to occur to a relatively low frequency with associated disorders identified during clinical assessments. On the contrary, dysgraphia appears linked to a high frequency of the associated disorders with a majority of oculomotor disorders (55% of children) leading to visual-perceptual difficulties (44%).

**Conclusions:**

HDs appear to be multifactorial but have a common characteristics of immaturity of gestural synergy of the arm, associated with poorer spatio-temporal kinetic and kinematic parameters. Dysgraphia occurs with more severe disorders as oculomotor and visual perception impairments. Our findings highlight the importance to identified a nosography of HDs with a transdisciplinary evaluation to better understand the nature and aetiology of the disorders in order to better clinical decision-making processes for handwriting remediations.

**Disclosure of Interest:**

None Declared

